# Neural Mechanisms of Prism Adaptation in Healthy Adults and Individuals with Spatial Neglect after Unilateral Stroke: A Review of fMRI Studies

**DOI:** 10.3390/brainsci11111468

**Published:** 2021-11-05

**Authors:** Olga Boukrina, Peii Chen

**Affiliations:** 1 Center for Stroke Rehabilitation Research, Kessler Foundation, West Orange, NJ 07052, USA; PChen@KesslerFoundation.org; 2 Department of Physical Medicine and Rehabilitation, New Jersey Medical School, Rutgers University, Newark, NJ 07103, USA

**Keywords:** spatial neglect, hemi-spatial neglect, stroke, fMRI, attention, prism adaptation

## Abstract

Functional disability due to spatial neglect hinders recovery in up to 30% of stroke survivors. Prism adaptation treatment (PAT) may alleviate the disabling consequences of spatial neglect, but we do not yet know why some individuals show much better outcomes following PAT than others. The goal of this scoping review and meta-analysis was to investigate the neural mechanisms underlying prism adaptation (PA). We conducted both quantitative and qualitative analyses across fMRI studies investigating brain activity before, during, and after PA, in healthy individuals and patients with right or left brain damage (RBD or LBD) due to stroke. In healthy adults, PA was linked with activity in posterior parietal and cerebellar clusters, reduced bilateral parieto-frontal connectivity, and increased fronto-limbic and sensorimotor network connectivity. In contrast, RBD individuals with spatial neglect relied on different circuits, including an activity cluster in the intact left occipital cortex. This finding is consistent with a shift in hemispheric dominance in spatial processing to the left hemisphere. However, more studies are needed to clarify the contribution of lesion location and load on the circuits involved in PA after unilateral brain damage. Future studies are also needed to clarify the relationship of decreasing resting state functional connectivity (rsFC) to visuomotor function.

## 1. Introduction

Among stroke survivors in the acute and subacute inpatient settings, approximately 30% have spatial neglect, which is more common after right than left brain damage (RBD and LBD, respectively) [1]. Spatial neglect can occur in individuals with other types of brain injury as well [2,3,4]. Spatial neglect is a neuropsychological syndrome that results from damage to the neural networks critical to the processing of spatial information and the control of attention [5,6]. The syndrome typically induces abnormal bias toward the space ipsilateral to the injured cerebral hemisphere, and hence, affected individuals pay insufficient or no attention to the contralesional side, which cannot be attributed to primary sensory or motor defects [7,8]. Symptoms of spatial neglect can be observed across domains (perception, representation, memory, movement planning, and motor control) and perceptual modalities (visual, auditory, tactile, proprioceptive) [9,10,11,12]. 

Spatial neglect has a significant clinical impact as it hinders rehabilitation progress and outcomes [13,14,15,16]. Prism adaptation treatment (PAT) is one of the interventions that can reduce symptoms of spatial neglect and improve functional outcomes [17,18]. However, the results of randomized sham-controlled trials in stroke patients are mixed with respect to unequivocal benefits of this treatment for all stroke patients [19,20,21,22,23]. It is unknown what determines the short-term beneficial effects and long-term therapeutic effects of PAT. In this article, we reviewed theoretical accounts for PAT mechanisms, conducted a series of meta-analyses of the available fMRI studies to determine what brain regions are activated before, during, and after prism adaptation, and built upon a framework that may improve the understanding of PAT effects on sensorimotor and cognitive plasticity as a way to augment recovery in patients with spatial neglect. 

### 1.1. A Brief History

**Figure 1:**
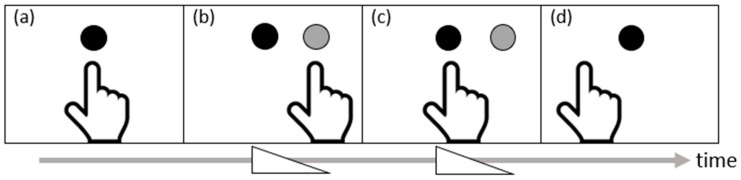
Prism adaptation illusion in four steps: (**a**) Before putting on prism lenses, the hand points to the target. (**b**) When putting on left-based prisms that induce a rightward shift of vision, the hand points to the shifted image instead of the actual target. (**c**) After several pointing attempts, the hand points to the actual target, which is left to the shifted image. This is prism adaptation. (**d**) Once the prism lenses are removed, the hand points to the left of the target. This is an after-effect.

Charles S. Harris was one of the first authors who conducted systematic experiments on prism adaptation. In his 1963 study, participants (with unspecified neurological background) were instructed to point to a central visual target 90 times while wearing wedged prism lenses that shifted the visual field to the left or right by approximately 11 degrees of visual angle. Harris observed the effects of prism adaptation after prism removal, i.e., after-effects, when asking participants to point to visual targets without seeing their arm and hand, to auditory targets with eyes closed, and straight ahead with eyes closed [24]. Participants erroneously pointed toward the side of space opposite to the lens shift (see Figure 1 for an illustration). Many replicated and expanded Harris’s observations in healthy individuals and found that the after-effects usually fade in a few minutes and are rarely detectable after about an hour depending on the measurement [25,26,27]. Nonetheless, what was learned from these early studies is that prism adaptation is a sensorimotor phenomenon that occurs without effortful strategy learning, and it temporarily alters motor behavior in a manner that is not necessarily determined by vision. 

More than three decades after Harris’s article (1963) was published, Yves Rossetti et al. [28] reported their application of prism adaptation in RBD individuals with left-sided neglect. Patients made 50 pointing movements to visual targets while wearing wedged prism lenses that shifted the visual field to the right by 10 degrees of visual angle (experimental condition) or flat thick lenses that induced no visual displacement (sham-control condition). Prism adaptation after-effects similar to those reported by Harris [24] were observed. More importantly, Rossetti et al. observed effects on neuropsychological tests sensitive to spatial neglect symptoms immediately after prism removal and two hours later [28]. This groundbreaking finding has inspired a new research area in the field of neurorehabilitation. Studies have focused on the visibility of pointing movements during adaptation [29,30], the degree of visual shift induced by prism lenses [31], the measurement of after-effects [32], and PAT effects on different frames of reference (more effective in egocentric than allocentric neglect) [33,34]. 

Many questions about PAT remain unanswered. We do not yet know how PAT effects expand from simple visuomotor tasks (e.g., reaching to a visual target) to functional tasks that may or may not be visuomotor in nature (e.g., grooming activities, text reading, postural balance) [35] and to tasks that are not visuomotor at all (e.g., mental imagery) [36]. The fact that patients can adapt to prisms (indicated by the presence of after-effects) and demonstrate improved symptoms of spatial neglect suggests that a common level of egocentric spatial representation is shared in sensorimotor adaptation, multi-modal and multi-domain integration, and higher-level cognitive spatial functions. Furthermore, while several studies investigated the neural mechanisms of a single session of prism adaptation (PA) and its after-effects [37,38,39], it is still unknown what brain areas support neuroplasticity associated with long-term effects of PAT, which subserve improvements in symptoms and severity of spatial neglect and in daily-life functions. To fill these knowledge gaps, it is important to understand the mechanisms of spatial neglect, of prism adaptation and its after-effects, and of the accumulated changes after multiple sessions of PAT. The present review is a first step toward a better understanding of the neuro-rehabilitative mechanisms of PAT and neuroplasticity associated with lasting improvement after PAT.

### 1.2. Neural Mechanisms of Spatial Neglect

Animal models of spatial neglect (rat, cat, and monkey) have revealed a well-characterized cortico-subcortical circuitry involved in directional orienting [40,41,42,43,44]. This circuitry includes cortico-striatal projections from visual and parietal areas to the superior colliculi and substantia nigra, as well as pathways linking the cerebellum and medial frontal/cingulate areas with brainstem oculomotor nuclei [40,41,44]. Unilateral lesions to cortical or collicular components of this circuit result in contralesional neglect. Such lesions upset the balance of activity between the two hemispheres, resulting in hyper-activation of the uninjured hemisphere. Consistent with the animal literature, spatial neglect in humans is associated with a partly overlapping set of lesions in parietal, frontal, temporal cortex, fronto-parietal white matter, corpus callosum, superior colliculi, caudate nucleus, and thalamus [44,45]. Although human data do not show the profound interhemispheric rivalry found in animals, there is evidence of hemispheric imbalance. Neglect is associated with abnormal dorsal and ventral parieto-frontal-temporal connectivity, intra- and interhemispheric parietal, frontal, and occipital disconnection, and a loss of segregation of functional neural networks within each hemisphere [46,47,48]. Accumulating evidence suggests that spatial neglect is not a “parietal cortex issue” or a “cortical disorder” but stems from a disruption of a widespread neural network.

### 1.3. Neural Mechanisms of Prism Adaptation (PA)

PA is a sensorimotor phenomenon achieved through two hypothetical stages of recalibration and realignment. Recalibration reflects the strategic adjustment of spatially coded movement commands aimed at rapidly reducing reaching errors, whereas realignment is a slower process of progressive remapping between visual and proprioceptive coordinate frames [49]. The neural bases of PA and its two stages have been studied in individuals with spatial neglect and in healthy participants using functional magnetic resonance imaging (fMRI). However, the findings are inconclusive. The paradigms used in these studies and their findings vary. For example, studies differed with respect to the types of prism goggles used during PA (neutral, leftward, or rightward deviating prisms, 5–20° shift of visual angle) and the nature of the pointing movement (e.g., finger pointing, laser pointing, or imaginary finger pointing), as well as at what timepoint brain activity was recorded (before, after, or during PA) and in which participants (healthy controls or stroke patients). Some studies in healthy individuals implicate the parietal cortex playing a key role in recalibration during the early phase of PA and the cerebellum in realignment of coordinate systems during the later phase of PA [50,51]. In contrast, some studies suggest the involvement of both the cerebellum and the posterior parietal areas in all phases of PA [51,52,53], with possible sub-specialization of parts of the cerebellum and parietal cortex (e.g., lobule VIII, IX involved in early strategic learning; lobule VI involved in later realignment) [53]. Other studies suggest that PA may alter the balance of activity in bilateral parietal, frontal, and temporal regions [37,54,55] and alters resting state fronto-parietal, parieto-temporal, and frontal-limbic connectivity [56]. In RBD patients with left-sided neglect, the effects of PA on brain activation differ from those in healthy participants and involve bilateral or left circuits [37,38]. In short, there is a growing pool of fMRI studies seeking to understand the neural mechanisms underlying PA and its after-effects. However, the results of these studies are mixed, which likely stems from differences in methodology and the subject population. 

### 1.4. Present Study

Based on the currently available evidence, we hypothesize that PA in healthy individuals relies on the right posterior parietal areas and bilateral cerebellum, with potentially different parts of the cerebellum engaged by early strategic recalibration and later progressive realignment of sensorimotor systems. Furthermore, among patients with spatial neglect, PA effects are hypothetically associated with one of two previously proposed processes: (1) a shift of hemispheric dominance within the ventral attentional system, which would restore the ventral attentional input to the dorsal attentional system via the intact contralesional occipito-temporal and inferior parietal cortex [57] or (2) an adjustment of the common reference frame for coordinated systems supported by parieto-cerebellar circuitry which serves to compensate for the failure of strategic setting of spatial parameters [58]. The aims of the present study were to examine the hypotheses stated above through a systematic evaluation of the published fMRI studies and to refine the theoretical framework for PA and PAT. 

## 2. Materials and Methods

We performed a scoping review and meta-analysis of the available literature on the neural bases of PA and PAT using fMRI. A PubMed search of “fMRI” and “prism adaptation” yielded a total of 50 published papers. We considered fMRI activation studies for cluster-based meta-analyses and resting state functional connectivity studies (rsFC) for qualitative review. The reason for excluding rsFC studies from the meta-analysis was that rsFC studies consider connectivity among pairs of brain regions or voxels, and thus, it was not possible to evaluate these studies using a cluster-based approach. Upon abstract review, 10 fMRI activation studies and 4 rsFC studies were selected. Following full text review, we eliminated 2 studies due to not having fMRI data or reviewing findings published elsewhere. Three additional fMRI studies were identified during the process of full text review. Each author conducted quality assessment of the included studies. 

A meta-analysis was performed on fMRI studies that shared common factors (e.g., participant population) if the number of studies was 2 or greater. For studies that did not meet this criterion, a comprehensive review of the studies was performed. We planned to conduct 4 cluster-based meta-analyses: (1) PA effects (pre-PA vs. post-PA) in healthy neurotypical participants (or healthy controls; HC), (2) PA effects in RBD patients, (3) PA mechanisms in HC using in-scanner PA, and (4) PA effects in LBD patients. In addition, we conducted 4 exploratory meta-analyses to study the neural correlates of PA separately for right deviating and left deviating prism goggles. A study could provide data in more than one category. Activation coordinates were extracted for each contrast related to prism exposure (pre > post) and to different PA phases (early vs. late). Whenever coordinates were not available from a published paper, we reached out to the corresponding author and requested them. A full list of activation coordinates is provided in the Supplementary Materials. All foci not already in the Montreal Neurological Institute (MNI) space were converted and submitted for analysis using the Brainmap GingerALE software version 3.0.2 [59,60]. A voxelwise thresholding of activation likelihood maps using family wise error (FWE) rate of 0.01 and 1000 permutations were used. The minimal cluster size threshold was set at 200 mm^3^.

## 3. Results 

### 3.1. Task-Specific fMRI Studies

We performed three meta-analyses instead of four because only one study [61] provided data related to LBD patients, which was, however, included in the qualitative evaluation in the context of our findings. Studies included in each meta-analysis are summarized in Table 1. Overall, a total of 171 foci from 32 contrasts collected from 616 participants (207 unique individuals, participating in repeated-measures experiments) were included.

#### 3.1.1. Meta-Analysis 1: Brain Activity before vs. after PA in HC

Five studies were included [37,54,55,61,62]. The meta-analysis included 97 foci from 13 contrasts and 391 participants (125 unique individuals participating in repeated-measures experiments). The meta-analysis yielded no significant clusters that survived the strict corrected thresholds. As this meta-analysis included studies that used both left and right deviating prism goggles, we carried out two additional exploratory meta-analyses to determine if any clusters were activated after as compared to before PA differentially for rightward vs. leftward PA. No clusters reached significance at the specified cluster threshold. However, if the threshold was lowered to a minimal cluster size of 150 mm^3^ and a FWE corrected *p* < 0.05 with 1000 permutations, we found that rightward PA was associated with an activation cluster in the left middle temporal gyrus with peak coordinates centered on x = −44, y = −66, z = 32, z = 5.25 (*p* < 0.05 FWE, cluster size = 168 mm^3^). This cluster was associated with an interaction of group (PA vs. control) and session (pre-PA vs. post-PA) and a contrast of post-PA > pre-PA for right targets in a visual detection task. Similarly, for leftward PA, no clusters were significant at the chosen threshold. When we decreased the cluster threshold to 150 mm^3^ and FWE corrected *p* < 0.05, two clusters became significant: a cluster in the right superior and middle temporal gyri (peak coordinates centered on x = 52, y = −58, z = 34, z = 5.72, *p* < 0.05 FWE, cluster size = 264 mm^3^) and a cluster in the left anterior cerebellum (peak coordinates centered on x = −27, y = −48, z = −23, z = 5.72, *p* < 0.05 FWE, cluster size = 160 mm^3^). The first cluster was associated with increased activation from pre- to post-PA to right stimuli in a visual detection task. The second cluster was linked with a decrease in activation post- compared to pre-PA to center and right stimuli in a visual detection task.

#### 3.1.2. Meta-Analysis 2: Brain Activity before vs. after PA in RBD Patients

**Figure 2:**
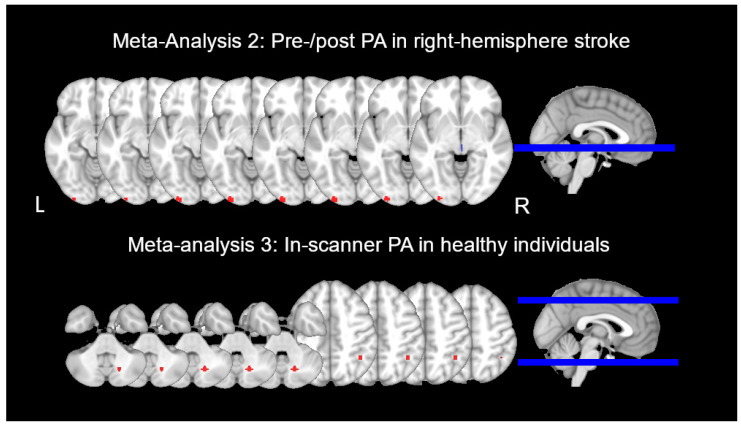
Significant clusters of activation identified using meta-analyses as implemented in Brainmap GingerALE software version 3.0.2 with a family-wise error rate of 0.01, 1000 permutations, and minimal cluster threshold of 200 mm^3^. Images are presented in neurological orientation; laterality is identified with letters L for left and R for right side of the brain.

Three studies were included [37,39,63]. The meta-analysis included 35 foci from seven contrasts and 69 participants (32 unique individuals participating in repeated-measures experiments). It identified a significant cluster of activation associated with the effect of PA. The cluster was in the left inferior occipital gyrus (IOG, BA 18) and posterior fusiform gyrus, with peak coordinates centered on x = −28, y = −98, z = −8 (z = 5.91, *p* < 0.01 FWE, cluster size = 393 mm^3^) (See Figure 2). The studies contributing to this cluster indicated that the cluster was active during line bisection and visual search to a greater degree after than before PA [39].

#### 3.1.3. Meta-Analysis 3: Progressive Change in Brain Activity during PA in HC

Four studies were included [50,51,53,64]. The meta-analysis included 39 foci from 12 contrasts and 156 participants (50 unique individuals participating in repeated-measures experiments). The meta-analysis identified two significant clusters of activation. The first cluster was in the right cerebellum, with a peak coordinate centered on x = 18, y = −66, z = −36 (z = 6.58, *p* < 0.01 FWE, cluster size = 392 mm^3^) (see Figure 2). The studies contributing to this cluster indicated that this cluster was active during both early and late PA but more active during the earlier period of PA [53]. The second cluster was found in the right inferior parietal lobule (IPL, BA 40), with a peak coordinate centered on x = 42, y = −50, z = 46 (z = 6.37, *p* < 0.01 FWE, cluster size = 264 mm^3^). The studies contributing to this cluster indicated that this cluster was active during both the early and the late phases of PA but was significantly more active during the later phase (e.g., [51]). Like in Meta-Analysis 1, we conducted two additional exploratory meta-analyses using the strict statistical threshold to separate the effects of rightward compared to leftward PA. Our results qualified the findings of the overall meta-analysis, suggesting that a large activation cluster in the right anterior cerebellum (peak coordinate centered on x = 18, y = −66, z = −36, z = 7.00, *p* < 0.01 FWE, cluster size = 584 mm^3^) was primarily linked with rightward PA, while an activation cluster in the right inferior parietal lobe (peak coordinate centered on x = 42, y = −50, z = 46, z = 7.04, *p* < 0.05 FWE, cluster size = 264 mm^3^) was primarily linked with leftward PA.

#### 3.1.4. PA Effects in LBD Patients 

Only one study considered in this review examined the effects of PA among LBD patients with right-sided neglect [61]. The study examined brain activity in the right hemisphere. In nine LBD patients, who performed an in-scanner visual detection task with left, center, and right targets, before and after wearing 10° leftward shifting prisms, a decrease in activation was observed when the stimuli were presented centrally and, in the left, non-neglected visual field. This decrease was found in four right hemisphere clusters, including (1) posterior middle and inferior temporal gyri (MTG, ITG) extending to IOG and middle occipital gyrus (MOG), (2) temporal pole, (3) the insula, and (4) a small cluster in the angular gyrus. The study also included 14 HC, in whom PA produced an increase in IPL activation to right stimuli, and a decrease in temporal and prefrontal pole activation to center and right stimuli. The differences in activation between stroke and healthy participants were not compared statistically.

### 3.2. Resting State Functional Connectivity (rsFC) fMRI Studies

The four rsFC studies (Table 2) were reviewed qualitatively [56,65,66,67]. These four published studies had three HC cohorts, asked different questions (rightward PA vs. control; rightward vs. leftward PA; before vs. after rightward PA), and used different connectivity analysis techniques (e.g., global connectivity, seed-based connectivity, correlation of connectivity strength with behavioral data). Studies employing seed-based functional connectivity analyses used bilateral intraparietal sulci (IPS), frontal eye fields (FEF), primary motor cortex (M1), cerebellum, left IPL, medial prefrontal cortex (*m*PFC), or anterior insula (*a*Ins) as seeds. Decreased connectivity was observed between the parietal seeds (IPS, left IPL) and bilateral superior temporal and inferior frontal cortices following PA, between the parietal seeds and bilateral cerebellum (following right PA) and additionally between the parietal seeds and right middle frontal, superior, and inferior parietal cortex following leftward PA (See Table 2). Similarly, connectivity of frontal seeds (FEF, *m*PFC) with IPS, right superior temporal sulcus (STS), left middle and right inferior frontal, left superior parietal areas and *a*Ins decreased following PA. Conversely, connectivity of the frontal seeds with anterior cingulate cortex (ACC) transitively increased following PA. Increased connectivity was also found between bilateral M1 seeds. IPS also increased connectivity with bilateral IPL and right middle frontal gyrus (MFG) following rightward PA. Thus, fronto-parietal connectivity decreased after PA, with one exception of increased IPS to IPL and MFG connectivity after rightward PA. Connectivity strength increased following PA within the motor cortex and between parietal seeds and the limbic cortex.

## 4. Discussion

PAT is an effective treatment for functional disabilities induced by spatial neglect [68]. However, despite ample evidence for PAT effectiveness, about 25% of studies fail to show the beneficial effects of PAT on daily function in individual participants with spatial neglect [17]. This conundrum is yet to be explained, as we do not fully understand the neural mechanisms underlying PAT responsiveness. Without such knowledge, our ability to maximize treatment effectiveness remains limited. In this review, we sought to elucidate the potential neuro-rehabilitative mechanisms of PAT by analyzing the currently available evidence for the neural mechanisms supporting PA. We employed qualitative and quantitative analytic approaches to determine what brain areas and brain networks become engaged before, during, and after PA, as evidenced by task-based and resting state fMRI studies in healthy adults and stroke patients with spatial neglect after unilateral brain damage. Our findings suggest that areas engaged during PA only partly overlap with areas that become more activated after PA. Moreover, the areas supporting PA effects among healthy controls differ from those among stroke patients, and the neural correlates of PA effects differ between RBD and LBD patients. While activity in most PA-supporting regions increases, rsFC among those regions predominantly decreases following PA. We review these findings below and conclude with a theoretical framework for interpreting these results in the context of what is known about oculomotor and attention networks.

### 4.1. Neural Mechanisms of Prism Adaptation in Healthy Adults 

Sensorimotor adaptation manipulated using optic prisms has fascinated generations of researchers [24,26,69,70,71]. Only in the recent decades did the fMRI technology enable the investigation of PA mechanisms at the brain level. We reviewed fMRI studies focused on neural activity and brain connectivity in healthy adults.

#### 4.1.1. Brain Activity before vs. after Prism Adaptation

Across the five studies [37,54,55,61,62] examined in Meta-Analysis 1 (Table 1), areas that became engaged in visual detection after PA, compared to before PA, included IPL, superior, middle, and inferior temporal gyri (STG, MTG, ITG), superior, middle, and inferior frontal gyri (SFG, MFG, IFG), insula, ACC, orbitofrontal and prefrontal cortex (OFC, PFC), cerebellum, postcentral gyrus, precuneus, and the occipital cortex. None of these cluster peaks survived multiple comparison correction in the planned meta-analysis, even though these five studies came from the same research group and employed a similar task. 

One reason for the null result of Meta-Analysis 1 may be that the regions identified were engaged either unilaterally or bilaterally, and the laterality often differed depending on the direction of prism shift (leftward vs. rightward PA, one study), modality (visual detection vs. auditory detection, one study), or stimulus location (left, right, or center, five studies). When we separated the analyses into contrasts examining rightward vs. leftward PA, we again failed to find any significant clusters at the specified cluster threshold. However, with a less conservative threshold, we observed increased activation of the contralateral STG/MTG after PA and decreased left cerebellum activation after PA in leftward PA studies. Luauté et al. [50] also observed activation of the STG/MTG associated with sustained PA when pointing errors were fully compensated and suggested that this activation is related to the generalization of spatial realignment to cognition rather than to PA per se. Chapman et al. [51] found sustained activation of the cerebellum bilaterally throughout the PA process; however, in their study, a more posterior part of the cerebellum was activated on the left in the early and late phases of PA. The role of the cerebellum is discussed further in the context of Meta-Analysis 3 findings.

Other factors that may have contributed to the heterogeneity of Meta-Analysis 1 results include a small number of participants for some of the studies reported (*n* < 15, three studies), suggesting that these studies may have only been powered to detect a two-way interaction between session (pre-PA and post-PA) and stimulus location (left, center, right) and not the main effect of the session. Different scanners, on which the data were collected, could have also contributed to the variability in the data. Lastly, the large number of brain regions found to be active in each study and included in the meta-analysis could have precluded any one region from being detected in the activation likelihood analysis. As the number of fMRI studies of PA grows in the future, it will become possible to subdivide these studies further into separate meta-analyses based on precise matches in methodology.

In contrast to activation studies, rsFC studies [56,65,66,67], based on a qualitative review, showed primarily a decrease in temporo-parietal, fronto-temporal, fronto-parietal, and parieto-cerebellar connectivity and an increase in parieto-limbic connectivity (transiently) and connectivity between bilateral motor seeds (Table 2). The global decreases in connectivity may be related to increased processing efficiency, which may or may not be specific to PA. Increased parieto-limbic (e.g., ACC) connectivity may be linked with increased arousal immediately following a PA session, which subsides 1 h after PA [56]. Our review also highlighted whether the finding that increased interhemispheric motor connectivity was associated with the size of the PA after-effects [65]. This result requires further research and the inclusion of behavioral covariates and additional control conditions. 

#### 4.1.2. Brain Activity during Prism Adaptation

Meta-Analysis 3 included some of the earliest landmark PA fMRI studies [50,51,53,64], which showed a circumscribed set of areas involved in online PA (Table 1). These areas include PPC, superior temporal cortex, and the cerebellum. While the superior temporal cortex is thought to support the transfer of PA effects to other cognitive domains as discussed earlier [51], there is some disagreement between the studies with respect to the specific role that PPC and the cerebellum play in PA. For example, Luauté et al. [50] reported that anterior IPL/IPS were engaged in the early phase of PA for recalibration, whereas cerebellum lobules IV and V were involved in the later phase of PA for the process of realignment. In contrast, Chapman et al. [51] implicated right angular gyrus in realignment, and Küper et al. [53] found that posterior cerebellum lobules VIII and IX were important for recalibration, whereas lobule VI was involved in realignment. Together, these studies had one thing in common: They had a relatively small number (<25) of visuomotor movements during PA in the scanner as compared to studies having PA outside the scanner (>50). As a result, the network of brain areas identified in these studies [50,51,53,64] is likely weighted toward earlier prism exposure effects. Thus, the results of Meta-Analysis 3 suggest that in healthy adults, PPC and the cerebellum play significant roles during PA, potentially, the early phase of PA (Figure 2). Specifically, among individuals who experience leftward PA (rightward after-effects), the mismatch between the visual input about target location and target-directed movement triggers rapid online correction of pointing errors and slower learning-based recalibration of visuomotor connectivity. Both processes are initially supported by the posterior cerebellum and involve cerebellar-parietal connectivity. Cerebellar activation subsides toward a later part of early PA, and recalibration is finalized through activity in the right IPL.

### 4.2. Neural Mechanisms of Prism Adaptation in Patients with Unilateral Stroke 

Post-stroke visuospatial deficits are associated with decreased connectivity of the ventral and dorsal fronto-parietal and temporal networks [6,46,48,72]. In contrast, increased functional connectivity and task-related activation of these networks is strongly coupled with spatial neglect recovery [47,73,74]. Much less understood are the neural bases of recovery associated with PAT (e.g., a treatment course of multiple sessions of PA) or the lack of improved outcomes at the deficit or functional level. There are mixed results in studies using similar protocols, e.g., 20-diopter lenses, one session a day for 10 sessions over two weeks [20,21,22,75]. Studies that doubled the sessions per day found positive effects in improving neglect [76] and rehabilitation outcomes [19]. Other trials reduced the course duration, such as one session a day over 4 days [77], or spaced out sessions, such as one to two sessions over 7–12 days [78], and improvements were limited on some but not all neuropsychological tests. Nonetheless, there is no prospective study looking into the neural mechanisms underlying PAT effects after a different number of sessions. Thus, we remain unable to determine the neural bases of recovery associated with PAT. However, growing evidence using different brain imaging and stimulation techniques offers credible hypotheses. 

Two hypotheses have been proposed to explain why rightward PA is able to alleviate the dysfunctional behaviors in left-sided spatial neglect among RBD patients. One hypothesis stated that PA facilitates compensation through the contribution of the intact left hemisphere [37,38]. The intact left IPL (part of the ventral attention system) receives input from the bilateral visual cortex. Following PA, hemispheric dominance within the ventral attention system is shifted to the left hemisphere (i.e., to the left rather than right IPL), which restores the input to bilateral dorsal attention system. This alleviates the pathological leftward orienting in spatial neglect [37]. Consistent with this theory, cathodal transcranial direct current stimulation (tDCS) applied over the left posterior parietal cortex interfered with the beneficial effect of PA on spatial neglect [79], while cortical thickness in left IPL and fusiform gyrus were positively associated with spatial neglect recovery after PA. Moreover, improvements of spatial neglect following 8 weeks of PAT were coupled with increased regional cerebral blood flow in parietal and pericallosal areas of the unaffected hemisphere [80]. Seemingly inconsistent with this hypothesis are the results of the longitudinal study by Nyffeler et al. [81] in RBD patients who were stimulated using theta-burst ‘inhibitory’ stimulation over the left parietal lobe with a generally positive response in clinical and neuropsychological symptoms of spatial neglect. This study suggests that inhibition of the left parietal cortex may be beneficial because it could restore putative transcallosal inhibition of the left by the right parietal cortex. However, given the importance of callosal integrity to this effect and, more generally, to spatial neglect recovery, Bartolomeo [82] argued that this result is also consistent with an improvement of spatial neglect through facilitation of compensatory interhemispheric communication. The perturbation introduced by continuous theta-burst stimulation may cause functional interhemispheric connectivity to shift from a pathologically isolated state toward a hub-like state of higher interconnectivity. This further highlights that inhibitory/excitatory stimulation effects may work in complex ways within bi-hemispheric brain networks.

The other hypothesis argued that spatial neglect represents a dysfunction of rapid calibration processes due to the abnormally reduced size of the task workspace where spatial attention is allocated [58]. Engaging in PA adjusts the position, but not the size, of this task workspace through a process of alignment, which represents a modification in transformational constants that help to translate between different sensory-motor systems. While calibration, which is responsible for error correction in PA, is accomplished through associative learning and is based in the supratentorial brain, alignment, responsible for PA after-effects, is subserved by the cerebellum [58]. Indeed, lesions in the cerebellum, along with those in the PPC, occipito-parietal, middle, and superior frontal cortex predict PA failure among spatial neglect patients [38].

Our finding in Meta-Analysis 2 showed that a cluster in the left inferior occipital/fusiform gyrus was more activated after than before PA in RBD patients (Figure 2) is in part consistent with the former hypothesis described above. Furthermore, this cluster was observed in studies that used visual search and line bisection tasks to index brain activity changes [39,63] and these tasks rely on visual processing. However, our results are inconsistent with either hypothesis, as we did not observe increased activation after PA in either the PPC or the cerebellum. While individual studies in this meta-analysis reported increases in IPL, SPL, SFG, MFG, and IFG, these increases were not consistent across studies, with either unilateral (left or right) or bilateral activity increases reported and only in some patient groups (frontal lesions patients as compared to parietal lesions patients). In contrast, all studies reported increased recruitment of bilateral occipital and fusiform cortex following PA. We propose that the main source of variability across these studies is likely the extent and location of stroke lesions given the small number of participants studied (N = 32 in the meta-analysis). The proportion of damage to the right inferior parietal area is also an important predictor of whether this area becomes recruited on the right or the left side of the brain. Future large-scale studies are needed to address these limitations.

Only one study in our review looked at brain activity before and after leftward PA among LBD patients [61] and was excluded from meta-analyses. The study was conducted by Crottaz-Herbette et al. who had conducted several similar studies in HC [37,54,55,61,62] and one in RBD patients [37]. While in LBD patients using leftward PA, primarily decreases of activity were observed in the right temporo-occipital and to some degree parietal areas to central and non-neglected left stimuli, in RBD patients using rightward PA, there were increases in the left temporo-parietal, bilateral occipital, and right frontal regions to stimuli located centrally or to either the left or right side. These findings are broadly consistent with the authors’ hypothesis that PA reverses right brain dominance in spatial attention [57]. In neurotypical healthy participants, right IPL is dominant and subserves spatial processing for both left and right visual field. Following a unilateral stroke and PA, the left hemisphere IPL either becomes more active, or right IPL becomes less active.

### 4.3. Theoretical Framework for Prism Adaptation

**Figure 3:**
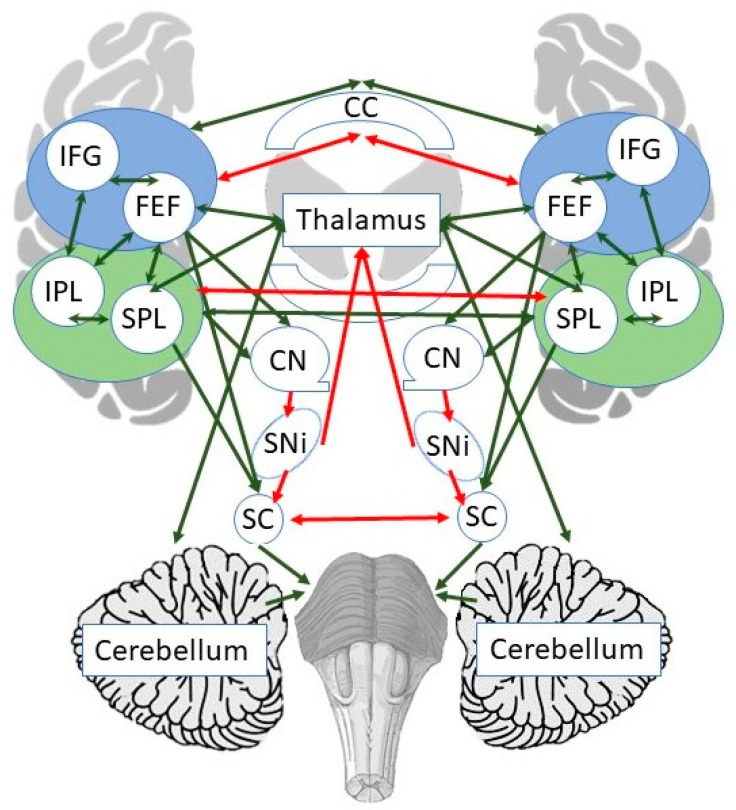
The anatomy of cortico-subcortical connectivity for oculomotor and attentional orienting, which may support prism adaptation. Inhibitory connections shown in red, excitatory connections shown in green. Blue circles denote frontal locations (goal-directed, intentional orienting). Green circles denote parietal locations (stimulus-driven, reflexive orienting). Not all possible connections are shown. CC—Corpus Callosum; CN—Caudate Nucleus; FEF—Frontal Eye Field; IPL—Inferior Parietal Lobule; SC—Superior Colliculus; SNi—Substantia Nigra; SPL—Superior Parietal Lobule.

Decades of animal and human work [40,41,42,43,44] resulted in a well-characterized cortical–subcortical network for oculomotor and attentional orienting, which includes dorsal, ventral frontal (FEF and IFG), and parietal (IPL, SPL) areas, as well at the thalamus, caudate nucleus, substantia nigra, superior colliculi, and the cerebellum (see Figure 3). Direct multisynaptic pathways connect the cortex and superior colliculi and have a net excitatory effect through multiple sign-inverting pathways, where the basal ganglia disinhibit the collicular neurons and contribute to the selection of desired movements (i.e., cerebral cortex → + Striatum → − Substantia Nigra → − Superior Colliculi). Right superior colliculus activates extraocular muscles and the left upper cervical motoneurons, either directly or through the reticular activating system, which induces head movement to the left and captures the stimulus in central vision. Simultaneously, there is a decrease in activity of the right cervical motoneurons, which allows the leftward head movement [40]. The cerebellum provides input to both the cortical and subcortical components of this network [83]. Together with sensorimotor networks for visually and proprioceptively guided movements, this system affords for PA [52]. 

**Figure 4:**
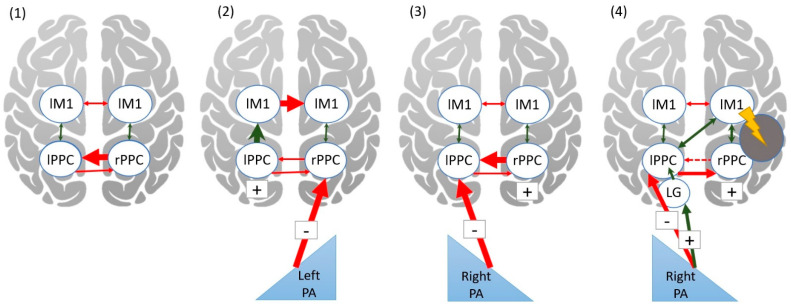
Hypothetical effects of PA in healthy adults (**1**–**3**) as proposed by Martín-Arévalo et al. (2018) [87] and in RBD patients (**4**). Inhibitory connections are shown in red; excitatory connections are shown in green. Under normal vision, right PPC inhibits the left. PA inhibits contralateral PPC. Following left PA, right PPC is inhibited, which disinhibits the left PPC and propagates activation to left M1, which in turn increases the inhibition of right M1. Following right PA, the inhibition of left PPC by right PPC is maintained. Following RBD, inhibition of left by right PPC is disrupted, the inhibition of left PPC by right PA is mediated by the left visual cortex, left PPC subsumes dominance in spatial orienting. M1—primary motor cortex, PPC—posterior parietal cortex, LG—lingual gyrus.

Our quantitative analyses of fMRI studies reinforce the key role of PPC and the cerebellum in the process of PA among healthy adults (Meta-Analysis 2) and the PA-induced reversal of right-brain dominance in visuospatial processing among RBD patients (Meta-Analysis 3). Our qualitative review of rsFC studies provides further insight into the modulating effects of PA. For example, one study reported interhemispheric connectivity changes between bilateral M1 seeds after PA, which were associated with the size of PA after-effects [65]. This is likely related to the presence of excitatory intrahemispheric functional connectivity between the PPC and M1 [84,85]. Similarly, a tDCS study showed that stimulation of the left motor cortex led to an increase in the size and retention of PA after-effects [86]. The authors of another study [87] proposed that PA exerts its after-effects by inhibiting the PPC contralateral to the prism deviation. They showed using transcranial magnetic stimulation (TMS) that PA altered interhemispheric inhibition of bilateral M1. The PA-induced inhibitory effect may modulate the inherent asymmetry between the left and right PPC [88]. The right PPC inhibits the left PPC, while the left does not inhibit the right to the same extent [88], which may account for a leftward bias in line bisection among some healthy adults (i.e., pseudoneglect) [89] (see Figure 4 panel 1). After a healthy individual adapts to leftward-PA and takes the prisms off, the net effect is reduced activity to visual targets in the right IPL, which disinhibits the left IPL. When asked to reach to a visual target, the excitatory connectivity between left IPL and M1 is enhanced due to the left IPL being disinhibited, and this also changes the bilateral M1 connectivity, which is proportionate to the strength of PA and the degree of left IPL disinhibition [65,87] (see Figure 4 panel 2). As a result, rightward after-effects as measured using arm-reaching pointing tasks are observed. The opposite prism shift may not trigger these processes to the same degree because transcallosal inhibitory effects in the PPC are asymmetric [88,90]. Thus, the right-to-left inhibition in PPC is maintained following rightward PA, and this frequently leads to an absence of any pointing after-effects in healthy adults [87,90] (see Figure 4 panel 3). 

However, after RBD, the right-to-left inhibition in PPC would be disrupted, and the PA mechanisms are supported by different circuits, which likely depend on lesion location [39]. In RBD patients with left-sided spatial neglect, rightward PA leads to leftward after-effects. Although rightward PA would normally inhibit the left IPL [88], in stroke patients, instead, it appears that the left hemisphere becomes more engaged. Some studies suggest that left parietal cortex subsumes dominance in spatial processing. Our meta-analysis suggests that greater left hemisphere recruitment following PA may involve the visual cortex, in particular, when behavioral tasks used to measure the effects of PA involve visual processing (Figure 4 panel 4). Thus, it is possible that in stroke patients, both sensorimotor realignment [58] (observed in HC, Meta-Analysis 3) and redistribution of hemispheric dominance [57] (Meta-Analysis 2) may take place at different phases of PA.

## 5. Conclusions

Based on our review and analyses of the available fMRI evidence about the neural mechanism of PA, healthy adults and individuals with spatial neglect after unilateral stroke adapt to prismatic exposure differently. In healthy adults, PA is carried out through cerebellar-parietal activation, which in turn affects the interhemispheric balance of activity in occipital, posterior parietal, and frontal primary motor areas. Patients with unilateral brain damage may rely on the intact hemisphere during PA; however, more studies are needed to clarify the contribution of lesion location and load on the circuits involved in PA. Similarly, future studies are needed to investigate the relationship of decreasing rsFC to visuomotor function. 

## Figures and Tables

**Table 1:** **Task-specific fMRI studies of prism adaptation (PA).** Meta-Analysis 1 includes five studies that scanned healthy individuals before and after PA who performed tasks different from the task they performed during PA. Meta-Analysis 2 includes three studies that scanned individuals with right brain damage (RBD) due to stroke before and after PA who performed the same or different tasks from the task they performed during PA. Meta-Analysis 3 includes four studies that scanned healthy individuals during PA.

**Meta-Analysis 1: Before and after PA in Healthy Individuals**							
**Study**	**Participant Population**	**N**	**PA Procedure**	**Task in Scanner**	**Scanned PA Phase**	**Findings**	**Quality Assessment**
Crottaz-Herbette et al., 2014 [55]	HC	28	10° rightward ≈150 concurrent finger pointing for 3 min, to left and right visual targets	Visual detection (pressing a button when a white stimulus appeared on black background), visuospatial short-term memory, verbal short-term memory	☒Before ☐Early ☐Late ☒After	Visual detection-- Group (PA > control) x session (after PA > before PA): ➢ Bilateral: IPL ➢ Left: SMG ➢ Right: AG PA group only, stimulus location (left, right, center) x session (after vs. before PA): ➢ Bilateral: IPL, SMG ➢ Left: mACC, ACC, AG, insula ➢ Right: IFG, MFG **Summary**: PA reversed right hemispheric dominance for visual space; left AG involvement in representation of the entire visual field after PA	**Strength**: Control group PA effects evaluated in a factorial model of whole brain activation. Varied stimulus position allowed investigation of PA effects in left, center, and right space
Crottaz-Herbette et al., 2017 [37]	Age-matched HC (only HC results noted here)	11	10° rightward ≈150 concurrent finger pointing for 3 min, to left and right visual targets	Visual detection (same as above)	☒Before ☐Early ☐Late ☒After	Stimulus location (left, right, center) x session (after vs. before PA) HC undergoing PA: ➢ Bilateral: STG, IPL, insula, SFG, MFG, IFG ➢ Left: MTG **Summary for HC**: Significant effect of PA in left hemisphere. PA in controls affected left TPJ, right temporal, and bilateral prefrontal cortex.	**Strength**: HC (age-matched) that were later compared to RBD patients PA effects evaluated in a factorial model of whole brain activation. Varied stimulus position allowed investigation of PA effects in left, center, and right space
Crottaz-Herbette et al., 2017 [54]	HC	42	10° rightward and leftward vs. 0° for neutral group ≈150 concurrent finger pointing for 3 min, to left and right visual targets	Visual detection (same as above)	☒Before ☐Early ☐Late ☒After	Group (l PA, r PA, neutral) x session (after vs. before PA) x stimulus location (left, right, center) ➢ Right: AG ➢ Left: ant. STG, MTG ➢ Bilateral: sup. med. parietal, precuneus, MFG, SMA, mid. CC Group (l PA, r PA, neutral) x session (after vs. before PA) ➢ Right: SMG ➢ Left: AG, MTG, MOG ➢ Bilateral: STG, OFC Group (l PA, r PA, neutral) effects after > before ➢ L-PA: rAG increased activity for right targets ➢ R-PA: lIPL increased activity for right, central, and left targets; rOFC, rMTG, bilat. MFG, lIFG increased activity for central and right targets ➢ Neutral: bilat. STG increased response to right targets; rSMG, bilat OC increased response to left targets	**Strength**: Systematic evaluation or right, left, and neutral PA PA effects evaluated in a factorial model of whole brain activation. Varied stimulus position allowed investigation of PA effects in left, center, and right space **Weakness**: No coordinates reported in the paper
Tissieres, et al., 2018 [62]	HC	30	10° rightward ≈150 concurrent finger pointing for 3 min, to left and right visual targets	Auditory detection and visual detection	☒Before ☐Early ☐Late ☒After	After vs. Before PA: ➢ Bilateral: insula, SMG, STG, PFC ➢ Left: AG, CBM, ➢ Right: MTG, precuneus Stimulus location (left, right, center) x session (after vs. before PA) Auditory detection group ➢ Bilateral: SMG, AG, IPL ➢ Left: SPL, precuneus, postcentral gyrus, STG, ITG Visual detection group: ➢ Bilateral: IPL, SMG ➢ Left: AG, MTG **Summary**: PA induced activation in left AG and cerebellum, bilateral insula, SMG, STG, and prefrontal cortex, and right MTG and precuneus irrespective of modality or stimulus position (right, center, left). Rightward PA enhanced representation of ipsilateral space within the left IPL and decreased it within the right IPL	**Strength**: PA effects evaluated in a factorial model of whole brain activation. Varied stimulus position allowed investigation of PA effects in left, center, and right space **Weakness**: Between-participant design. Only 16 participants completed the auditory task, and 14 participants completed the visual task, limiting the sample size for each task Data acquired on different MRI scanners and scanner effect not taken into consideration
Crottaz-Herbette et al., 2019 [61]	HC	14	10° leftward 150 concurrent finger pointing (1–1.5 sec between pointings), to left and right visual targets	Visual detection (same as that used in their 2014 study)	☒Before ☐Early ☐Late ☒After	After vs. Before PA: ➢ rIPL increased activity to right stimuli ➢ rTP, rPFC, activity decreased activity to left, right, and center stimuli	**Strength**: PA effects evaluated in a factorial model of whole brain activation. Varied stimulus position allowed investigation of PA effects in left, center, and right space **Weakness**: HCs not matched for age of LBD patients in the same study No statistical analysis between HC and LBD groups Sample size too small to examine the difference between patients with and without right-sided neglect In patients, only effects in the right (intact) hemisphere were considered
**Meta-Analysis 2: Before and after PA in individuals with RBD**							
Saj et al., 2013 [63]	RBD	7	20° rightward 50 concurrent finger pointing to left and right visual targets presented above the MRI bed	“bisection” (conceptually similar to a landmark task), visual search, and spatial memory	☒Before ☐Early ☐Late ☒After	“bisection”-- After > before PA: ➢ Bilateral: MFG, SPL, OC Visual search-- After > before PA: ➢ Bilateral: MFG, SPL, OC ➢ Right: TPJ **Summary**: Reduction of left neglect after PA associated with bilateral increases in SPL, MFG, occipital lobe	**Strength**: Using the same visual stimuli in different tasks Reporting individual differences in behavioral data
Saj et al., 2019 [39]	RBD	10	20° rightward Imaginary finger pointing and encouraged rapid saccades to left and right visual targets	Imaginary finger pointing and encouraged rapid saccades to visual target	☒Before ☒Early ☒Late ☒After	Frontal group Early PA: ➢ Left: CBM, OC Mid PA: ➢ Right: CBM, OC Late PA: ➢ Bilateral: OC, PPC ➢ Right: CBM Bisection, After > before PA: ➢ Bilateral: PC, PFC, OC Search, After > before PA: ➢ Bilateral: FC ➢ Left: PC, TC Parietal group Early PA: ➢ Left: CBM, OC Mid PA: ➢ Right: CBM, OC Late PA: ➢ Bilateral: CBM, OC ➢ Left: PC Bisection, After > before PA: ➢ Bilateral: OC Search, After > before PA: ➢ Bilateral: OC **Summary**: Increased activity in bilateral fronto-parietal networks and the occipital lobe following PA treatment, larger increases in patient group with frontal than parietal lesions	**Strength**: Patients with specific lesion locations **Weakness**: Imaginary PA may not recruit the same neural activities as normal PA
Crottaz-Herbette et al., 2017 [37]	RBD	15	10° rightward ≈150 concurrent finger pointing for 3 min, to left and right visual targets	Visual detection (same as that used in their 2014 study above)	☒Before ☐Early ☐Late ☒After	Stimulus location (left, right, center) x session (after vs. before PA) Bilateral: hippocampus, thalamus, parahippocampal, fusiform, precuneus and lingual gyri, mCG, paracentral lobule, calcarine, aCBM, vermis Left: SPL, IPL, STG, SMG, STG, AG, SFG, MFG, IFG **Summary**: PA in patients affected IPL, prefrontal, and temporal cortex. Anterior STG/MTG activity correlated with neglect severity, with greater increases after PA for more severe spatial neglect.	**Strength**: Age-matched healthy controls PA effects evaluated in a factorial model of whole brain activation. Varied stimulus position allowed investigation of PA effects in left, center, and right space
**Meta-Analysis 3: During PA in healthy individuals**							
Chapman et al., 2010 [51]	HC	12	10° leftward Laser pointing to visual target appearing at one of two left locations or two right locations Monocular PA	Laser pointing to visual target while viewing it through a monocular lens	☒Before ☒Early ☒Late ☒After	Early PA > “baseline” (pointing but no laser): ➢ Left: pCBM ➢ Right: aCBM, SPL, IPL, SMG Late PA > “baseline” (pointing but no laser): ➢ Bilateral: pCBM ➢ Right: aCBM, SPL, aIPL Late > early PA: ➢ Right: aIPL, AG, pCBM **Summary**: Anterior IPL and cerebellum activated during early and late phase of PA	**Weakness**: Upper limb invisible during PA, which differs drastically from conventional procedures No comparison of after vs. before PA ROI-based analysis using ROI from a functional localizer, did not consider full brain No fMRI data related to after-effects were reported although participants stayed in the scanner and performed tasks after PA
Danckert et al., 2008 [64]	HC	8	5° (10 diopter) rightward 10 concurrent finger pointing to left and right visual targets Monocular PA	Finger pointing to visual target while viewing through a monocular prism lens during the ON condition	☐Before ☒Early ☒Late ☐After	Early PA > late PA: ➢ Left: primary motor, aCG, aIPL ➢ Right: medial CBM **Summary**: Anterior cingulate, anterior intraparietal sulcus and right medial cerebellum activated early during PA	**Weakness**: No motion correction Only 10 pointing trials during PA and a long delay (11.5 s) between trials (the reasoning of ON and OFF conditions in early and late trials is unclear) After-effects unknown Pointing performance not measured during PA
Küper et al., 2014 [53]	HC	19	20° rightward Terminal finger pointing to left and right visual targets Monocular PA	Finger pointing to visual target while viewing through a monocular lens	☒Before ☒Early ☒Late ☒After	Before, early, late, and after PA: ➢ Bilateral: aCBM ➢ Right: pCBM, vermis, medial and lateral CBM Good > bad adapters during late PA: ➢ Bilateral: lobule VI ➢ Right: lobules V **Summary**: Strategic learning in PA associated with ventro-caudal dentate and posterior cerebellum activity; spatial realignment associated with superior posterior cerebellum	**Strength**: High-resolution (7T) scanner **Weakness**: Only examined the cerebellum
Luauté et al., 2009 [50]	HC	11	10° leftward 24 concurrent finger pointing to left and right visual targets Monocular PA	Finger pointing to visual target while viewing through a monocular lens	☒Before ☒Early ☒Late ☒After	Early and late PA > before PA: ➢ Bilateral: STS, STG Early > late PA: ➢ Bilateral: IPS, SPL ➢ Right: CBM lobules IV and V After > before PA: ➢ Left: IPL **Summary**: PA activated STG/STS; Early PA activated right cerebellum, SPL, IPS, left IPS. Activity in anterior IPS modulated by error size; de-adaptation activated left IPL	**Strength**: Analysis results compared to visuo-motor control network mask (pointing vs. button press)

Note: ° = degrees of visual angle shift by prism lenses. Abbreviations: ACC = anterior cingulate cortex, AG = angular gyrus; a = anterior; CBM = cerebellum; CG = cingulate gyrus; FC = frontal cortex; IFG = inferior frontal gyrus; IOG = inferior occipital gyrus; IPL = inferior parietal lobe; l = left hemisphere; med = medial; MFG = middle frontal gyrus; mid = middle; MOG = middle occipital gyrus; N = sample size; OC = occipital cortex; OFC = orbito-frontal cortex; p = posterior; PA = prism adaptation; PC = parietal cortex; PFC= prefrontal cortex; r = right hemisphere; RBD = right brain damage; SMG = supramarginal gyrus; SPL = superior parietal lobule; STG = superior temporal gyrus; TP = temporal pole; TPJ = temporo-parietal junction.

**Table 2:** **Resting-state fMRI (rs-fMRI) studies of prism adaptation (PA).** Four studies are included. Participants were scanned before and after PA. PA was conducted outside of scanner.

Study	Participant Population	N	PA Procedure	Task in Scanner	Findings	Quality Assessment
Schintu et al., 2020 [67]	HC	38 (18 used rightward prisms; 20 used leftward prisms)	15° rightward or leftward 150 concurrent finger pointing movements to right and left visual targets verbally cued in pseudorandom order	Looking at a white central cross appearing on a black screen for an unspecified period of time	R ight vs. left PA in decreasing rsFC between IPS seeds and … Right PA: ➢ A cluster of right parahippocampal, fusiform and lingual gyri, and Thal ➢ Left and right CBM Left PA: No effect survived FDR correction After vs. before PA in rsFC between IPS seeds and … Right PA ➢ Bilateral (decreased): STG ➢ Bilateral (increased): IPL ➢ Right (increased): MFG Left PA ➢ Bilateral (decreased): STG ➢ Right (decreased): MFG, SPL ➢ Left (decreased): IPL	**Strength**: Adequate sample size; direct comparison of left vs. right PA **Weakness**: Connectivity analyses only examined two seeds
Tsujimoto et al., 2019 [56]	HC	19	11.4° rightward (using 20-diopter prism lenses) 90 terminal finger pointing movements to central, right, and left visual targets randomized on a monitor screen	10 min with unspecified instructions	Immediately after PA vs. before in rsFC between FEF and … ➢ Right (decreased): IPS ➢ Right (increased): ACC One hour after PA vs. immediate after PA in rsFC between FEF and … ➢ Right (increased): IPS ➢ Right (decreased): ACC	**Strength**: Second fMRI, one hour after PA, allowed evaluating the duration of connectivity changes **Weakness**: Narrow field of view (four slices) Analyses limited to connectivity between specific, pre-determined seeds Instructions to participants in scanner were not described
Tsujimoto et al., 2019 [65]	HC	19	11.4° rightward (using 20-diopter prism lenses) 90 terminal finger pointing movements to central, right, and left visual targets randomized on a monitor screen	10 min with unspecified instructions	Before and after PA, observable increases in rsFC were found among four seeds: bilateral M1 and bilateral CBM Correlation between PA after-effect and rsFC change: right and left M1	**Strength**: Correlating behavioral data (PA after-effect) and rsFC change **Weakness**: Narrow field of view (four slices) Focused on before and after PA and did not analyze data collected one hour after PA
Wilf et al., 2019 [66]	HC	26 (14 in the PA group; 12 in the control group)	10° rightward 150 concurrent finger pointing to left and right visual targets	Looking at a red fixation cross for 8 min	After vs. before PA in the PA group (n = 14) Global connectivity (GC) decreased among: ➢ Bilateral: mPFC ➢ Left: aInsula, IPL Seed-based connectivity decreased between left IPL and … ➢ Left: aInsula ➢ Right: STS, IFG Seed-based connectivity decreased between right mPFC and … ➢ Left: aInsula and DAN areas in the parietal and frontal cortex ➢ Right: STS, IFG Seed-based connectivity decreased between left aInsula and … ➢ Left: precuneus, IPL ➢ Right: mPFC	**Strength**: Both global and seed-based connectivity changes were considered **Weakness**: Seed-based connectivity analysis only considered seeds significant in the global connectivity analysis, which may create inflated effects No direct comparison between the PA and control groups in rs-fMRI analyses

## Data Availability

Data used in the present study were extracted from published articles and are available in the Supplementary Materials.

## Supplementary Materials

The following are available online at https://www.mdpi.com/article/10.3390/brainsci11111468/s1, Activation Coordinates Used in the Meta-Analyses.

## References

[1] Esposito E., Shekhtman G., Chen P. (2021). Prevalence of spatial neglect post stroke: A systematic review. Ann. Phys. Rehabil. Med..

[2] Chen P., Ward I., Khan U., Liu Y., Hreha K. (2016). Spatial neglect hinders success of inpatient rehabilitation in individuals with traumatic brain injury: A retrospective study. Neurorehabil. Neural. Repair..

[3] Gomes D., Fonseca M., Garrotes M., Lima M.R., Mendonca M., Pereira M., Lourenco M., Oliveira E., Lavrador J.P. (2017). Corpus callosum and neglect syndrome: Clinical findings after meningioma removal and anatomical review. J. Neurosci. Rural. Pract..

[4] Brain W.R. (1941). Visual disorientation with special reference to lesions of the right cerebral hemisphere. Brain.

[5] Mesulam M.M. (1999). Spatial attention and neglect: Parietal, frontal and cingulate contributions to the mental representation and attentional targeting of salient extrapersonal events. Philos. Trans. R. Soc. Lond. B. Biol. Sci..

[6] Corbetta M., Shulman G.L. (2011). Spatial neglect and attention networks. Annu. Rev. Neurosci..

[7] Heilman K.M., Valenstein E. (1979). Mechanisms underlying hemispatial neglect. Ann. Neurol..

[8] Heilman K.M., Watson R.T., Valenstein E., Heilman K.M., Valenstein E. (2012). Neglect and related disorders. Clinical Neuropsychology.

[9] Adair J.C., Barrett A.M. (2008). Spatial neglect: Clinical and neuroscience review: A wealth of information on the poverty of spatial attention. Ann. N. Y. Acad. Sci..

[10] Harvey M., Rossit S. (2012). Visuospatial neglect in action. Neuropsychologia.

[11] Ogourtsova T., Archambault P., Lamontagne A. (2015). Impact of post-stroke unilateral spatial neglect on goal-directed arm movements: Systematic literature review. Top. Stroke Rehabil..

[12] Rode G., Pagliari C., Huchon L., Rossetti Y., Pisella L. (2017). Semiology of neglect: An update. Ann. Phys. Rehabil. Med..

[13] Katz N., Hartman-Maeir A., Ring H., Soroker N. (1999). Functional disability and rehabilitation outcome in right hemisphere damaged patients with and without unilateral spatial neglect. Arch. Phys. Med. Rehabil..

[14] Wee J.Y., Hopman W.M. (2008). Comparing consequences of right and left unilateral neglect in a stroke rehabilitation population. Am. J. Phys. Med. Rehabil..

[15] Chen P., Hreha K., Kong Y., Barrett A.M. (2015). Impact of spatial neglect in stroke rehabilitation: Evidence from the setting of an inpatient rehabilitation facility. Arch. Phys. Med. Rehabil..

[16] Yoshida T., Mizuno K., Miyamoto A., Kondo K., Liu M. (2020). Influence of right versus left unilateral spatial neglect on the functional recovery after rehabilitation in sub-acute stroke patients. Neuropsychol. Rehabil..

[17] Champod A.S., Frank R.C., Taylor K., Eskes G.A. (2018). The effects of prism adaptation on daily life activities in patients with visuospatial neglect: A systematic review. Neuropsychol. Rehabil..

[18] Chen P., Diaz-Segarra N., Hreha K., Kaplan E., Barrett A.M. (2021). Prism adaptation treatment improves inpatient rehabilitation outcome in individuals with spatial neglect: A retrospective matched control study. Arch. Rehabil. Res. Clin. Transl..

[19] Mizuno K., Tsuji T., Takebayashi T., Fujiwara T., Hase K., Liu M. (2011). Prism adaptation therapy enhances rehabilitation of stroke patients with unilateral spatial neglect: A randomized, controlled trial. Neurorehabil. Neural. Repair..

[20] Ten Brink A.F., Visser-Meily J.M.A., Schut M.J., Kouwenhoven M., Eijsackers A.L.H., Nijboer T.C.W. (2017). Prism adaptation in rehabilitation? No additional effects of prism adaptation on neglect recovery in the subacute phase poststroke: A randomized controlled trial. Neurorehabil. Neural. Repair..

[21] Vilimovsky T., Chen P., Hoidekrova K., Petioky J., Harsa P. (2021). Prism adaptation treatment to address spatial neglect in an intensive rehabilitation program: A randomized pilot and feasibility trial. PLoS ONE.

[22] Serino A., Barbiani M., Rinaldesi M.L., Ladavas E. (2009). Effectiveness of prism adaptation in neglect rehabilitation: A controlled trial study. Stroke.

[23] Li J., Li L., Yang Y., Chen S. (2021). Effects of prism adaptation for unilateral spatial neglect after stroke: A systematic review and meta-analysis. Am. J. Phys. Med. Rehabil..

[24] Harris C.S. (1963). Adaptation to displaced vision: Visual, motor, or proprioceptive change?. Science.

[25] Taub E., Goldberg L.A. (1973). Prism adaptation: Control of intermanual transfer by distribution of practice. Science.

[26] Redding G.M., Wallace B. (1978). Sources of “overadditivity” in prism adaptation. Percept. Psychophys..

[27] Redding G.M., Wallace B. (2008). Intermanual transfer of prism adaptation. J. Mot. Behav..

[28] Rossetti Y., Rode G., Pisella L., Farne A., Li L., Boisson D., Perenin M.T. (1998). Prism adaptation to a rightward optical deviation rehabilitates left hemispatial neglect. Nature.

[29] Ladavas E., Bonifazi S., Catena L., Serino A. (2011). Neglect rehabilitation by prism adaptation: Different procedures have different impacts. Neuropsychologia.

[30] Facchin A., Bultitude J.H., Mornati G., Peverelli M., Daini R. (2020). A comparison of prism adaptation with terminal versus concurrent exposure on sensorimotor changes and spatial neglect. Neuropsychol. Rehabil..

[31] Facchin A., Beschin N., Toraldo A., Cisari C., Daini R. (2013). Aftereffect induced by prisms of different power in the rehabilitation of neglect: A multiple single case report. Neurorehabilitation.

[32] Sarri M., Greenwood R., Kalra L., Papps B., Husain M., Driver J. (2008). Prism adaptation aftereffects in stroke patients with spatial neglect: Pathological effects on subjective straight ahead but not visual open-loop pointing. Neuropsychologia.

[33] Gossmann A., Kastrup A., Kerkhoff G., Lopez-Herrero C., Hildebrandt H. (2013). Prism adaptation improves ego-centered but not allocentric neglect in early rehabilitation patients. Neurorehabil. Neural. Repair..

[34] Mancuso M., Damora A., Abbruzzese L., Zoccolotti P. (2018). Prism adaptation improves egocentric but not allocentric unilateral neglect: A case study. Eur. J. Phys. Rehabil. Med..

[35] Jacquin-Courtois S., O’Shea J., Luaute J., Pisella L., Revol P., Mizuno K., Rode G., Rossetti Y. (2013). Rehabilitation of spatial neglect by prism adaptation: A peculiar expansion of sensorimotor after-effects to spatial cognition. Neurosci. Biobehav. Rev..

[36] Rode G., Rossetti Y., Boisson D. (2001). Prism adaptation improves representational neglect. Neuropsychologia.

[37] Crottaz-Herbette S., Fornari E., Notter M.P., Bindschaedler C., Manzoni L., Clarke S. (2017). Reshaping the brain after stroke: The effect of prismatic adaptation in patients with right brain damage. Neuropsychologia.

[38] Lunven M., Rode G., Bourlon C., Duret C., Migliaccio R., Chevrillon E., Thiebaut de Schotten M., Bartolomeo P. (2019). Anatomical predictors of successful prism adaptation in chronic visual neglect. Cortex.

[39] Saj A., Cojan Y., Assal F., Vuilleumier P. (2019). Prism adaptation effect on neural activity and spatial neglect depend on brain lesion site. Cortex.

[40] Payne B.R., Rushmore R.J. (2004). Functional circuitry underlying natural and interventional cancellation of visual neglect. Exp. Brain Res..

[41] Payne B.R., Rushmore R.J. (2003). Animal models of cerebral neglect and its cancellation. Neuroscientist.

[42] Burcham K.J., Corwin J.V., Stoll M.L., Reep R.L. (1997). Disconnection of medial agranular and posterior parietal cortex produces multimodal neglect in rats. Behav. Brain Res..

[43] Sprague J.M. (1966). Interaction of cortex and superior colliculus in mediation of visually guided behavior in the cat. Science.

[44] Valero-Cabre A., Toba M.N., Hilgetag C.C., Rushmore R.J. (2020). Perturbation-driven paradoxical facilitation of visuo-spatial function: Revisiting the ’Sprague effect’. Cortex.

[45] Wiesen D., Karnath H.O., Sperber C. (2020). Disconnection somewhere down the line: Multivariate lesion-symptom mapping of the line bisection error. Cortex.

[46] Baldassarre A., Ramsey L., Hacker C.L., Callejas A., Astafiev S.V., Metcalf N.V., Zinn K., Rengachary J., Snyder A.Z., Carter A.R. (2014). Large-scale changes in network interactions as a physiological signature of spatial neglect. Brain.

[47] He B.J., Snyder A.Z., Vincent J.L., Epstein A., Shulman G.L., Corbetta M. (2007). Breakdown of functional connectivity in frontoparietal networks underlies behavioral deficits in spatial neglect. Neuron.

[48] Toba M.N., Migliaccio R., Batrancourt B., Bourlon C., Duret C., Pradat-Diehl P., Dubois B., Bartolomeo P. (2018). Common brain networks for distinct deficits in visual neglect. A combined structural and tractography MRI approach. Neuropsychologia.

[49] Prablanc C., Panico F., Fleury L., Pisella L., Nijboer T., Kitazawa S., Rossetti Y. (2020). Adapting terminology: Clarifying prism adaptation vocabulary, concepts, and methods. Neurosci. Res..

[50] Luaute J., Schwartz S., Rossetti Y., Spiridon M., Rode G., Boisson D., Vuilleumier P. (2009). Dynamic changes in brain activity during prism adaptation. J. Neurosci..

[51] Chapman H.L., Eramudugolla R., Gavrilescu M., Strudwick M.W., Loftus A., Cunnington R., Mattingley J.B. (2010). Neural mechanisms underlying spatial realignment during adaptation to optical wedge prisms. Neuropsychologia.

[52] Panico F., Rossetti Y., Trojano L. (2020). On the mechanisms underlying prism adaptation: A review of neuro-imaging and neuro-stimulation studies. Cortex.

[53] Kuper M., Wunnemann M.J., Thurling M., Stefanescu R.M., Maderwald S., Elles H.G., Goricke S., Ladd M.E., Timmann D. (2014). Activation of the cerebellar cortex and the dentate nucleus in a prism adaptation fMRI study. Hum. Brain Mapp..

[54] Crottaz-Herbette S., Fornari E., Tissieres I., Clarke S. (2017). A brief exposure to leftward prismatic adaptation enhances the representation of the ipsilateral, right visual field in the right inferior parietal lobule. eNeuro.

[55] Crottaz-Herbette S., Fornari E., Clarke S. (2014). Prismatic adaptation changes visuospatial representation in the inferior parietal lobule. J. Neurosci..

[56] Tsujimoto K., Mizuno K., Nishida D., Tahara M., Yamada E., Shindo S., Kasuga S., Liu M. (2019). Prism adaptation changes resting-state functional connectivity in the dorsal stream of visual attention networks in healthy adults: A fMRI study. Cortex.

[57] Clarke S., Crottaz-Herbette S. (2016). Modulation of visual attention by prismatic adaptation. Neuropsychologia.

[58] Redding G.M., Wallace B. (2006). Prism adaptation and unilateral neglect: Review and analysis. Neuropsychologia.

[59] Turkeltaub P.E., Eickhoff S.B., Laird A.R., Fox M., Wiener M., Fox P. (2012). Minimizing within-experiment and within-group effects in Activation Likelihood Estimation meta-analyses. Hum. Brain Mapp..

[60] Eickhoff S.B., Laird A.R., Grefkes C., Wang L.E., Zilles K., Fox P.T. (2009). Coordinate-based activation likelihood estimation meta-analysis of neuroimaging data: A random-effects approach based on empirical estimates of spatial uncertainty. Hum. Brain Mapp..

[61] Crottaz-Herbette S., Tissieres I., Fornari E., Rapin P.A., Clarke S. (2019). Remodelling the attentional system after left hemispheric stroke: Effect of leftward prismatic adaptation. Cortex.

[62] Tissieres I., Fornari E., Clarke S., Crottaz-Herbette S. (2018). Supramodal effect of rightward prismatic adaptation on spatial representations within the ventral attentional system. Brain Struct. Funct..

[63] Saj A., Cojan Y., Vocat R., Luaute J., Vuilleumier P. (2013). Prism adaptation enhances activity of intact fronto-parietal areas in both hemispheres in neglect patients. Cortex.

[64] Danckert J., Ferber S., Goodale M.A. (2008). Direct effects of prismatic lenses on visuomotor control: An event-related functional MRI study. Eur. J. Neurosci..

[65] Tsujimoto K., Mizuno K., Nishida D., Tahara M., Yamada E., Shindo S., Watanabe Y., Kasuga S., Liu M. (2019). Correlation between changes in functional connectivity in the dorsal attention network and the after-effects induced by prism adaptation in healthy humans: A dataset of resting-state fMRI and pointing after prism adaptation. Data Brief.

[66] Wilf M., Serino A., Clarke S., Crottaz-Herbette S. (2019). Prism adaptation enhances decoupling between the default mode network and the attentional networks. Neuroimage.

[67] Schintu S., Freedberg M., Gotts S.J., Cunningham C.A., Alam Z.M., Shomstein S., Wassermann E.M. (2020). Prism adaptation modulates connectivity of the intraparietal sulcus with multiple brain networks. Cereb. Cortex.

[68] Yang N.Y., Zhou D., Chung R.C., Li-Tsang C.W., Fong K.N. (2013). Rehabilitation interventions for unilateral neglect after stroke: A systematic review from 1997 through 2012. Front. Hum. Neurosci..

[69] Harris C.S. (1965). Perceptual adaptation to inverted, reversed, and displaced vision. Psychol. Rev..

[70] Redding G.M., Wallace B. (2006). Generalization of prism adaptation. J. Exp. Psychol. Hum. Percept. Perform..

[71] Held R., Schlank M. (1959). Adaptation to disarranged eye-hand coordination in the distance-dimension. Am. J. Psychol..

[72] Barrett A.M., Boukrina O., Saleh S. (2019). Ventral attention and motor network connectivity is relevant to functional impairment in spatial neglect after right brain stroke. Brain Cogn..

[73] Corbetta M., Kincade M.J., Lewis C., Snyder A.Z., Sapir A. (2005). Neural basis and recovery of spatial attention deficits in spatial neglect. Nat. Neurosci..

[74] Thimm M., Fink G.R., Sturm W. (2008). Neural correlates of recovery from acute hemispatial neglect. Restor. Neurol. Neurosci..

[75] Goedert K.M., Chen P., Foundas A.L., Barrett A.M. (2020). Frontal lesions predict response to prism adaptation treatment in spatial neglect: A randomised controlled study. Neuropsychol. Rehabil..

[76] Priftis K., Passarini L., Pilosio C., Meneghello F., Pitteri M. (2013). Visual scanning training, limb activation treatment, and prism adaptation for rehabilitating left neglect: Who is the winner?. Front. Hum. Neurosci..

[77] Nys G.M.S., de Haan E.H.F., Kunneman A., de Kort P.L.M., Dijkerman H.C. (2008). Acute neglect rehabilitation using repetitive prism adaptation: A randomized placebo-controlled trial. Restor. Neurol. Neurosci..

[78] Vaes N., Nys G., Lafosse C., Dereymaeker L., Oostra K., Hemelsoet D., Vingerhoets G. (2018). Rehabilitation of visuospatial neglect by prism adaptation: Effects of a mild treatment regime. A randomised controlled trial. Neuropsychol. Rehabil..

[79] Ladavas E., Giulietti S., Avenanti A., Bertini C., Lorenzini E., Quinquinio C., Serino A. (2015). a-tDCS on the ipsilesional parietal cortex boosts the effects of prism adaptation treatment in neglect. Restor. Neurol. Neurosci..

[80] Shiraishi H., Yamakawa Y., Itou A., Muraki T., Asada T. (2008). Long-term effects of prism adaptation on chronic neglect after stroke. Neurorehabilitation.

[81] Nyffeler T., Vanbellingen T., Kaufmann B.C., Pflugshaupt T., Bauer D., Frey J., Chechlacz M., Bohlhalter S., Muri R.M., Nef T. (2019). Theta burst stimulation in neglect after stroke: Functional outcome and response variability origins. Brain.

[82] Bartolomeo P. (2019). Visual neglect: Getting the hemispheres to talk to each other. Brain.

[83] Baumann O., Borra R.J., Bower J.M., Cullen K.E., Habas C., Ivry R.B., Leggio M., Mattingley J.B., Molinari M., Moulton E.A. (2015). Consensus paper: The role of the cerebellum in perceptual processes. Cerebellum.

[84] Chao C.C., Karabanov A.N., Paine R., Carolina de Campos A., Kukke S.N., Wu T., Wang H., Hallett M. (2015). Induction of motor associative plasticity in the posterior parietal cortex-primary motor network. Cereb. Cortex.

[85] Karabanov A.N., Chao C.C., Paine R., Hallett M. (2013). Mapping different intra-hemispheric parietal-motor networks using twin Coil TMS. Brain Stimul..

[86] O’Shea J., Revol P., Cousijn H., Near J., Petitet P., Jacquin-Courtois S., Johansen-Berg H., Rode G., Rossetti Y. (2017). Induced sensorimotor cortex plasticity remediates chronic treatment-resistant visual neglect. eLife.

[87] Martin-Arevalo E., Schintu S., Farne A., Pisella L., Reilly K.T. (2018). Adaptation to leftward shifting prisms alters motor interhemispheric inhibition. Cereb. Cortex.

[88] Koch G., Cercignani M., Bonni S., Giacobbe V., Bucchi G., Versace V., Caltagirone C., Bozzali M. (2011). Asymmetry of parietal interhemispheric connections in humans. J. Neurosci..

[89] Jewell G., McCourt M.E. (2000). Pseudoneglect: A review and meta-analysis of performance factors in line bisection tasks. Neuropsychologia.

[90] Goedert K.M., Leblanc A., Tsai S.W., Barrett A.M. (2010). Asymmetrical effects of adaptation to left- and right-shifting prisms depends on pre-existing attentional biases. J. Int. Neuropsychol. Soc..

