# Supplementary material for: Neural Mechanisms of Prism Adaptation in Healthy Adults and Individuals with Spatial Neglect after Unilateral Stroke: A Review of fMRI Studies
Source: Brain Sci. 2021 Nov 5;11(11):1468. doi: 10.3390/brainsci11111468 (PMC8615640; doi:10.3390/brainsci11111468)
Supplement: Supplementary file 1 [file brainsci-11-01468-s001.zip › brainsci-1411964-supplementary.pdf]

**Activation Coordinates Used in the Meta-Analyses (Montreal Neurological Institute space)**

1. Meta-Analysis 1: Pre-/post PA in healthy individuals

//Crottaz-Herbette et al., 2014: 2-way interaction of group x session visual detection task

//Subjects=28

| | | |
|---|---|---|
| 44 | -30 | 26 |
| -46 | -66 | 32 |

//Crottaz-Herbette et al., 2014: 2-way interaction of group x session visuospatial short term memory task

//Subjects=28

| | | |
|---|---|---|
| -28 | 52 | -2 |
| 52 | 10 | 42 |
| -12 | -70 | -26 |
| 14 | -44 | 34 |
| 16 | -72 | 44 |
| -28 | -58 | 44 |
| -28 | 4 | -16 |
| 18 | -52 | 68 |
| -4 | -84 | -10 |
| -34 | -86 | 0 |

//Crottaz-Herbette et al., 2017: 2-way interaction of stimulus position (left, right, center) x session (pre-PA, post-PA)

//Subjects=11

| | | |
|---|---|---|
| -56 | -26 | 0 |
| -42 | 42 | 2 |
| 50 | 20 | 8 |
| -42 | -62 | 38 |

| | | |
|---|---|---|
| 66 | -16 | 10 |
| -14 | 44 | 50 |
| -38 | 18 | 50 |
| 32 | 42 | -14 |
| 40 | -52 | 42 |
| 24 | 40 | 48 |

//Crottaz-Herbette, 2017 (eNeuro): 3-way interaction of group (lPA, rPA, neurtral) x session (pre-PA, post-PA) x stimulus position (L, R, C) on visual detection task

//Subjects=42

| | | |
|---|---|---|
| 2 | 52 | -18 |
| -8 | -70 | 34 |
| -32 | 34 | 46 |
| 46 | -78 | 30 |
| -2 | -16 | 34 |
| 30 | 12 | 60 |
| -50 | 2 | -18 |

//Crottaz-Herbette et al., 2017b: 2-way interaction of group (lPA, rPA, neurtral) x session (pre-PA, post-PA) on visual detection task

//Subjects=42

| | | |
|---|---|---|
| -30 | -2 | -14 |
| 42 | -30 | 24 |
| 54 | 2 | -4 |
| -44 | 22 | 44 |
| 46 | 40 | -16 |
| -26 | -58 | -24 |
| 8 | -78 | 32 |
| -28 | -32 | -10 |

| | | |
|---|---|---|
| -42 | 32 | -4 |
| -40 | -70 | -10 |
| -48 | -66 | 24 |

//Crottaz-Herbette et al, 2017b: paired t-test of post>pre-left PA right targets on visual detection task

//Subjects=42

| | | |
|---|---|---|
| 52 | -58 | 34 |
| 8 | 44 | -10 |

//Crottaz-Herbette et al., 2017b:  paired t-test post>pre-right PA for right targets on visual detection task

//Subjects=42

| | | |
|---|---|---|
| -44 | -66 | 30 |

//Crottaz-Herbette et al., 2017b:  paired t-test post>pre-right PA for central targets on visual detection task

//Subjects=42

| | | |
|---|---|---|
| 50 | 42 | -16 |
| -48 | -52 | 44 |

//Crottaz-Herbette et al., 2017b: paired t-test post>pre-right PA for left targets on visual detection task

//Subjects=42

| | | |
|---|---|---|
| -48 | -74 | 36 |
| -52 | 6 | 28 |
| 0 | -42 | 34 |
| 18 | -74 | 40 |
| 32 | 18 | 48 |
| 36 | 40 | -12 |

| | | |
|---|---|---|
| 46 | -54 | 8 |
| -2 | 42 | -16 |
| 60 | -18 | -22 |
| -2 | 22 | 48 |

//Tissieres et al., 2018: 2-way interaction of stimulus position (left, right, center) x session (pre-PA, post-PA), combined auditory and visual tasks

//Subjects=30

| | | |
|---|---|---|
| 42 | -50 | 52 |
| -48 | -46 | 46 |
| 32 | 18 | 46 |
| 32 | -66 | -12 |
| -36 | -50 | -16 |
| -40 | 30 | 20 |
| 36 | 20 | -4 |
| -38 | 22 | 0 |
| -6 | -72 | 46 |
| 4 | -64 | 54 |
| 28 | -8 | -20 |

//Crottaz-Herbette et al., 2019: increase post vs. pre-PA to right stimuli on visual detection task

//Subjects=14

| | | |
|---|---|---|
| 52 | -58 | 34 |

//Crottaz-Herbette et al., 2019: decrease post vs. pre-PA to right stimuli on visual detection task

//Subjects=14

| | | |
|---|---|---|
| 30 | -38 | 0 |
| 50 | 12 | 18 |

| | | |
|---|---|---|
| -24 | -38 | 8 |
| 48 | -44 | 8 |
| -28 | -48 | -24 |
| 58 | -38 | 44 |
| -40 | -62 | -8 |
| -34 | 18 | -6 |
| 48 | 38 | 2 |
| -38 | 0 | -18 |
| -16 | -72 | 52 |
| -60 | -38 | 30 |
| -36 | -32 | 12 |
| -24 | 4 | 58 |
| -42 | 50 | 6 |

//Crottaz-Herbette et al., 2019: decrease post vs. pre-PA to center stimuli on visual detection task

//Subjects=14

| | | |
|---|---|---|
| -40 | 30 | -6 |
| 30 | 16 | -8 |
| -26 | -48 | -22 |
| 38 | -8 | 66 |
| -10 | 36 | 14 |
| -24 | 44 | 18 |
| 44 | -70 | -8 |
| 48 | -24 | 38 |
| -36 | -16 | 38 |
| 56 | -60 | 14 |
| -48 | -30 | 52 |

| | | |
|---|---|---|
| -6 | -76 | 26 |
| 60 | -20 | -6 |
| -14 | -38 | 40 |
| -12 | -2 | 70 |

2. Meta-Analysis 2: Pre-/post PA in individuals with right-hemisphere stroke

//Saj et al., 2013: after prism > before prism line bisection task

//Subjects=7

| | | |
|---|---|---|
| -24 | -60 | 73 |
| 22 | -61 | 66 |
| -34 | 21 | 42 |
| 24 | 37 | 36 |
| -34 | -97 | -10 |
| 24 | -94 | -18 |

//Saj et al., 2013: after prism > before prism visual search task

//Subjects=7

| | | |
|---|---|---|
| -8 | -63 | 74 |
| 31 | -60 | 76 |
| -40 | 12 | 53 |
| 37 | 25 | 47 |
| -25 | -100 | -10 |
| 34 | -102 | 3 |
| 57 | -35 | 23 |

//Crottaz-Herbette et al., 2017: 2-way interaction of stimulus position (left, right, center) x session (pre-PA, post-PA)

//Subjects=15

| | | |
|---|---|---|
| -18 | -38 | 4 |
| -30 | -56 | 56 |
| -36 | 26 | -6 |
| 4 | -24 | 50 |
| -12 | 52 | 42 |
| -40 | 12 | 40 |
| -46 | -78 | 28 |
| -62 | -46 | 24 |

//Saj et al., 2019: Line Bisection post>pre-PA, frontal lesion group

//Subjects=10

| | | |
|---|---|---|
| -48 | -67 | 40 |
| 40 | -58 | 49 |
| -23 | 22 | 49 |
| 30 | 11 | 46 |
| -30 | -97 | -11 |
| 18 | -100 | 1 |

//Saj et al., 2019: Line Bisection post>pre-PA, parietal lesion group

//Subjects=10

| | | |
|---|---|---|
| -30 | -94 | -2 |
| 12 | -70 | 10 |

//Saj et al., 2019: visual search post>pre-PA, frontal lesion group

//Subjects=10

| | | |
|---|---|---|
| -45 | -70 | 37 |
| -54 | -70 | 28 |
| -14 | 23 | 60 |
| 32 | 41 | 34 |

//Saj et al., 2019: visual search post>pre-PA, parietal lesion group

//Subjects=10

-27 -97 -5

15 -88 1

3. Meta-Analysis 3: In-scanner PA in healthy individuals

//Danckert, Ferber, & Goodale, 2008: Prisms on vs. prisms off more active during first 3 pointing trials than during last 3

//Subjects=8

-36 -16 44

2 -1 49

-39 -40 51

1 -62 -4

//Luauté et al., 2009: sustained PA exposure, run1 + run2 of PA vs. run1 x 2

//Subjects=11

58 -12 -12

70 -20 4

-60 -26 4

// Luauté et al., 2009: early vs. late PA exposure

//Subjects=11

24 -38 -32

40 -54 66

-30 -68 40

// Luauté et al., 2009: PA exposure, areas negatively correlated with error size

//Subjects=11

-16 -66 40

// Luauté et al., 2009: PA exposure, areas positively correlated with error size

//Subjects=11

-46 -54 56

// Luauté et al., 2009: De-adaptation: pointing baseline pre vs. post-PA

//Subjects=11

-48 -58 52

//Chapman et al., 2010: error correction< spatial realignment

//Subjects=12

16 -58 -50

36 -78 30

42 -50 46

//Chapman et al., 2010: error correction > baseline no visual feedback condition

//Subjects=12

-20 -64 -24

16 -50 -20

14 -62 60

42 -50 46

//Chapman et al., 2010: spatial realignment > baseline no visual feedback condition

//Subjects=12

-20 -64 -24

16 -50 -20

| | | |
|---|---|---|
| 16 | -58 | -50 |
| 14 | -62 | 60 |
| 42 | -50 | 46 |
| 36 | -78 | 30 |

//Küper et al., 2014: early PA vs. baseline visuomotor pointing without prisms

//Subjects=19

| | | |
|---|---|---|
| 31 | -56 | -52 |
| 31 | -49 | -51 |
| 13 | -67 | -31 |
| 18 | -66 | -36 |

//Küper et al., 2014: late PA vs. rest

//Subjects=19

| | | |
|---|---|---|
| 3 | -60 | -19 |
| -5 | -48 | -25 |
| -10 | -54 | -34 |
| 8 | -56 | -29 |
| 18 | -65 | -39 |

//Küper et al., 2014: early vs. late PA

//Subjects=19

| | | |
|---|---|---|
| 31 | -57 | -52 |
| 6 | -62 | -42 |
| 13 | -67 | -31 |
| 18 | -66 | -36 |
